# Vaccination—Its Uses and Abuses

**Published:** 1889-06

**Authors:** Edward Haughton


TO THE EDITOR OF HALL'S JOURNAL OF HEALTH :

### VACCINATION—ITS USES AND ABUSES.

BY EDWARD HAUGHTON, M. D.

### 1ST. WHAT IS VACCINATION.

Before any rational decision as to the advantages or disadvantages of vaccination can possibly be arrived at, another question must be answered without dissimulation, namely, "What is vaccination ?" This question may be answered by the impudent assertion that "everybody knows that," or by endeavoring to raise some side issue so as to leave the questioner without any satisfactory answer. We may be referred back to the days of Jenner and regaled with the interesting story of his conversation with a milkmaid and his notion about horse grease, swine pox and the like. But the question now is this : If I am desirous of being vaccinated with the best vaccine lymph, what is the nature and source of the animal virus which will probably be provided for me ? I may, of course, shut my eyes and open my mouth, as is sometimes said to children when sweetmeats are offered them. But vaccine "lymph is not a sweetmeat," neither is it a "natural secretion like milk and butter," as was once said by a quandom president of the British Medical Association. It is something which Sir James Paget assures us is very "beneficial" though it produces a permanent morbid condition of the blood. According to my conception of the proper usage of words, what is called "vaccine lymph" is not lymph at all, but a fluid excreted from a sore produced by an animal virus, whose exact nature is the subject of much controversy amongst leading members of the medical profession and which must continue to be the subject of controversy so long as it is derived from sources altogether different from one another.

Dr. Jenner stated that the injection employed by him came, in the first instance, from the horse originating in a complaint called "the grease," which is attended by an oozing of thin matter from the heels of the animal ; and this, according to Mr. Seaton's evidence before a parliamentary committee in 1871, constitutes the principal supply for the United Kingdom. As the results of using this lymph have been severely criticised on all sides, and the public confidence in its efficacy begins to wane, it has been seen by the advocates of the Compulsory Vaccination Acts, that unless some substitute could be found in which the public could still believe, the entire repeal of those acts with all their emoluments would follow.

### HOW TO AVOID VACCINATION.

The correspondence in the press from medical men and from parents, whose children have been severely and fatally injured by vaccination, compels the confession that vaccination is a hazardous operation, and unfortunately it does not prevent or mitigate smallpox. It produces fever and ill health at the most delicate period of life, and is the cause of much suffering and many infantile deaths. Bearing these facts in mind, I have thought it advisable to instruct parents how to avoid the dreaded operation. A bad law must be resisted, or it will never be got rid of. Those who can afford, should fight the law step by step. When vaccination notices are sent in they should be disregarded, if a summons follows, put every obstacle in the way of the officers, and if fined, refuse to pay the fine, make your goods over to your wife, and let them take you to prison. Those who have leisure can in this way serve the truth and the cause of public health. When you come out of gaol, an indignation meeting should be convened, and the fallacies of the repulsive rite exposed. Others may safeguard their offspring by paying the judicial penalties, for the law does not compel forcible vaccination. Those who are unable to pay fines, may act thus. Before the child is born, let the mother go to the house of some friend or relation for her confinement, register the child from her temporary abode, and then return home. The vaccination officer will be put off the track of the parent, and the child escapes. The plan of not registering the child (resorted to by many) cannot be recommended. The poorer classes may adopt a different course. When the vaccination notice comes, give your landlord notice to quit, and quietly remove into other lodgings. By these means you may save your offspring from lasting injury. Vaccination officers are sometimes "squared," and giving the officer half a crown on the quiet may save you a much larger sum in doctor's bills. Many towns have anti-vaccination societies and "defense funds," you pay a subscription of about five shillings a year, and for this ruin or imprisonment is often averted. If summoned, some one may appear for you, and if you are fined, the society pays your fines. If all these means fail, go to a respectable doctor and ask him to vaccinate your baby in one place, and make as small a scar as he can. If he refuses, take the baby to another doctor, as many are cognizant of the perils of vaccination who will gladly carry out your instructions. The doctor is not bound to vadcinate with calf or human lymph, but may vaccinate with water, glycerine or tartar emetic. If you feel obliged to go tɔ the vaccination station, refuse to have your child done in more than one place. If the doctor tries to do more you must resist, and tell him you will have as little mischief done as possible. If he is resolved to make more marks make a disturbance, a doctor dislikes nothing so much as this, as your determination to protect your baby, will excite sympathy for you and indignation against him. Lastly, take with you a bit of linen rag that has been soaked in a little borax and water, and as soon as you leave the vaccinating surgeon, with this wash off the virus. This neutralizes the poison and prevents injury, or the lymph may be soaked out. When baby has been operated on two or three times, the doctor will fill in your paper, as not susceptible.

At all hazards, save your defenceless children and show your neighbors these instructions, so they may save theirs.

### VACCINATION UNPHILOSOPHICAL—A MEDICAL WITNESS.

I hold it unphilosophical either to ignore or attempt to sneer down the invincible facts so conclusively adduced *against* the practice of compulsory vaccination. Assuredly they have as much valuable scientific evidence in them as any facts that can be found in practical medicine. Enough of itself to condemn *enforced* vaccination is the certainty of the introduction of other and worse diseases *sooner or later* whatever care be exercised. Vaccination was at first proclaimed an absolute preventive of smallpox. No vaccinator now ventures on this absurd falsehood. At the present time vaccination, it is asserted, mitigates smallpox and prevents its mortality. Universal facts afford world-wide evidence that vaccine virus neither prevents smallpox nor its fatality.

According to half a century's medical experience in hospital and private practice, I can testify, AS A PUBLIC VACCINATOR, that incalculable benefit to humanity would at once result if enforced "cowpox" were abandoned.

WILLIAM HITCHMAN, M. R. C. P. (England).

